# “Different trend” in multiple primary lung cancer and intrapulmonary metastasis

**DOI:** 10.1186/s40001-015-0109-5

**Published:** 2015-02-20

**Authors:** Cheng Shen, Xin Wang, Long Tian, Yubin Zhou, Dali Chen, Heng Du, Weiya Wang, Lunxu Liu, Guowei Che

**Affiliations:** Department of Thoracic Surgery, West-China Hospital, Sichuan University, Chengdu, 610041 China; Department of Pathology, West-China Hospital, Sichuan University, Chengdu, 610041 China

**Keywords:** Primary metachronous lung cancer, Metastatic lung cancer, Microsatellite analysis

## Abstract

**Background:**

The distinguishing of intrapulmonary metastatic tumors from multiple primary lung cancers is difficult but of great importance for the therapeutic management and prognosis of these patients.

**Methods:**

We used genomic DNA analyzed by six microsatellites (D7S1824, D15S822, D2S1363, D10S1239, D6S1056, and D22S689) with PCR to identify discordant allelic variation from 12 patients. There are five patients with multiple primary lung cancers and seven patients who were diagnosed with intrapulmonary metastases from 850 patients with primary lung cancer in our hospital. The experiments were approved by the West China Hospital Ethics committee (No. 2013 (33)) and all patients agreed to participate in the study and signed an informed consent form.

**Results:**

In the group of metachronous lung tumor, three of five patients have different histological types and one of five patients have the same histological type which showed “contradictory trend”. The other one showed “unique trend”. In the second group (intrapulmonary metastasis lung tumor), one patient showed “contradictory trend” and the others showed “unique trend”.

**Conclusions:**

“Different trends” are useful in discrimination of intrapulmonary metastasis lung cancer and multiple primary lung cancer even diagnosed with the histopathological evaluation.

## Background

It is known that patients with pulmonary neoplasms are at an increased risk for a second tumor in the lung, either at the same time or later in their life [1-3]. Dual primary lung cancer refers to two or more primary cancers in different sites of one or both lungs, with either consistent or different histology but no association between two cancers. Based on the time when the tumors are identified, the disease can be classified as synchronous or metachronous [4]. The incidence of multiple primary lung cancers has been reported to range from 0.7% to 15% of patients with lung cancer [5-9]. The frequency of recorded synchronous or metachronous lung cancers has been increasing in the recent years because of the development of early detection techniques, such as computed tomography (CT) and positron emission tomography (PET), and advances in cancer therapy. Although surgical treatment can also be a curative option for multiple primary lung cancers as well as single lung cancer, the 5-year survival of patients with synchronous multiple primary lung cancers has been reported to range from 0% to 44% [10,11] despite an early diagnosis. One of the reasons for such a wide range in this survival data is that patients who were clinically diagnosed with multiple primary lung cancers may include some patients with intrapulmonary metastasis. At present, the majority of multiple primary lung cancers are misdiagnosed as metastatic cancer in the issue based on pathology and radiology. Especially, when patients develop multiple, morphologically similar lung cancer, the clinical diagnosis becomes critical for the selection of an appropriate treatment. In the absence of carcinoma *in situ*, the morphologic similarity between the first and the second lung neoplasm can make it impossible to make the diagnostic distinction between metachronous primary neoplasms and solitary pulmonary metastasis. Thus, traditional histopathological assessment of neoplasms of the aerodigestive tract cannot definitively distinguish multiple primary cancers from metastatic disease when solitary, histologically similar cancers arise synchronously or metachronously in an individual patient.

Recent advances in molecular biology have provided several markers that can be used for clonal analysis [12-14]. Allelic variation between neoplasms often reflects accumulation of differential chromosomal deletion events [15]. In our previous study, we analyzed genomic instability expression profiles in 18 patients, whose histologies indicated that they were primary synchronous lung cancers, with ten cases of pulmonary tumors with metastasis (to the brain, sternum, or adrenal gland) selected for comparison. We found that, with polymorphic microsatellite markers, the “unique trend” that represents metastasis cancers and the “contradictory trend” that represents primary multiple tumors are useful in the diagnosis of synchronous multiple lung tumors [16].

In this study, we used a PCR-based approach to screen polymorphic microsatellite markers as a discrimination marker of double primary lung cancers from intrapulmonary metastasis. We examined five patients with metachronous multiple lung tumors and seven patients diagnosed with intrapulmonary metastasis for their variations in allele numbers. The results support the mechanism of “different trends” and illustrate the potential power of molecular techniques in differential diagnosis.

## Methods

### Patients and clinical features

**Figure 1:**
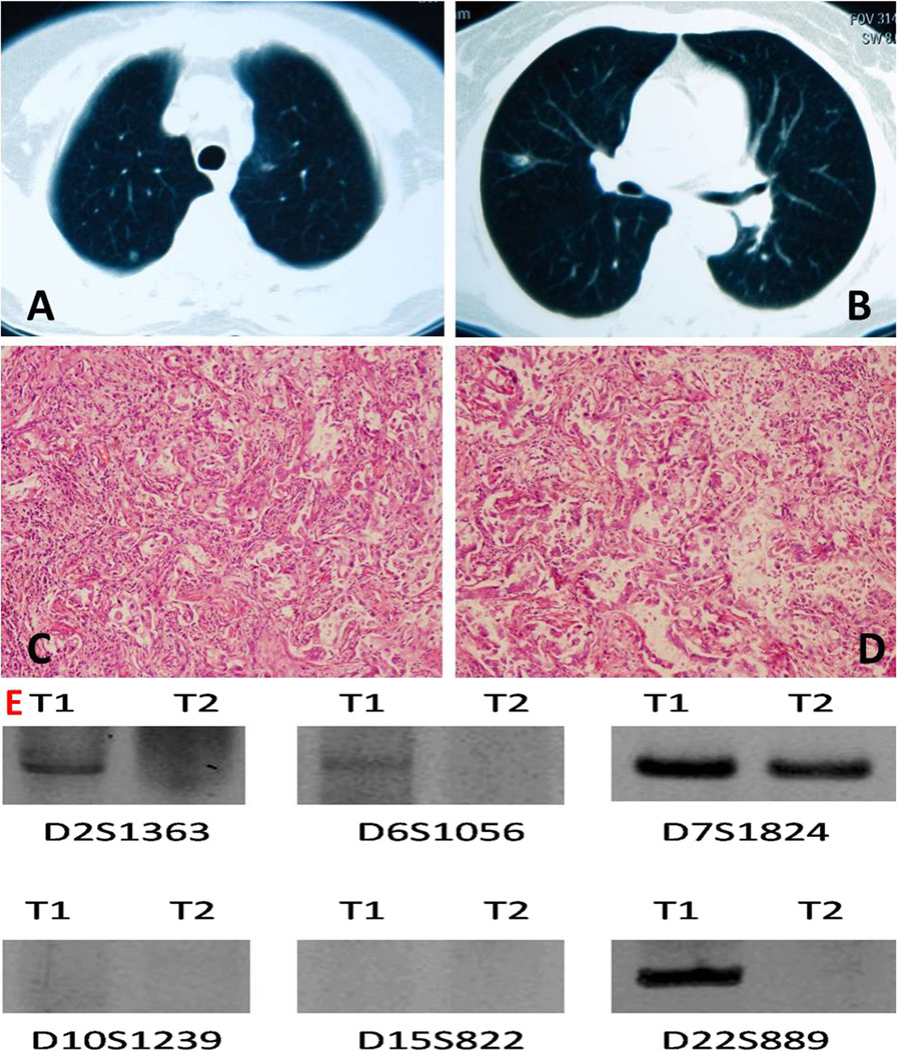
**CT features, histological features, and molecular analysis of patient 8 in the intrapulmonary metastasis group. A** and **B**: the first tumor and the second tumor of patient 8. **C** and **D**: two tumors of patient 8 in the intrapulmonary metastasis group were diagnosed pathologically as adenocarcinoma (H & E staining X100). **E**: T1 refers to the tumor in the right upper lobe, and T2 refers to the tumor in the right middle lobe.

**Figure 2:**
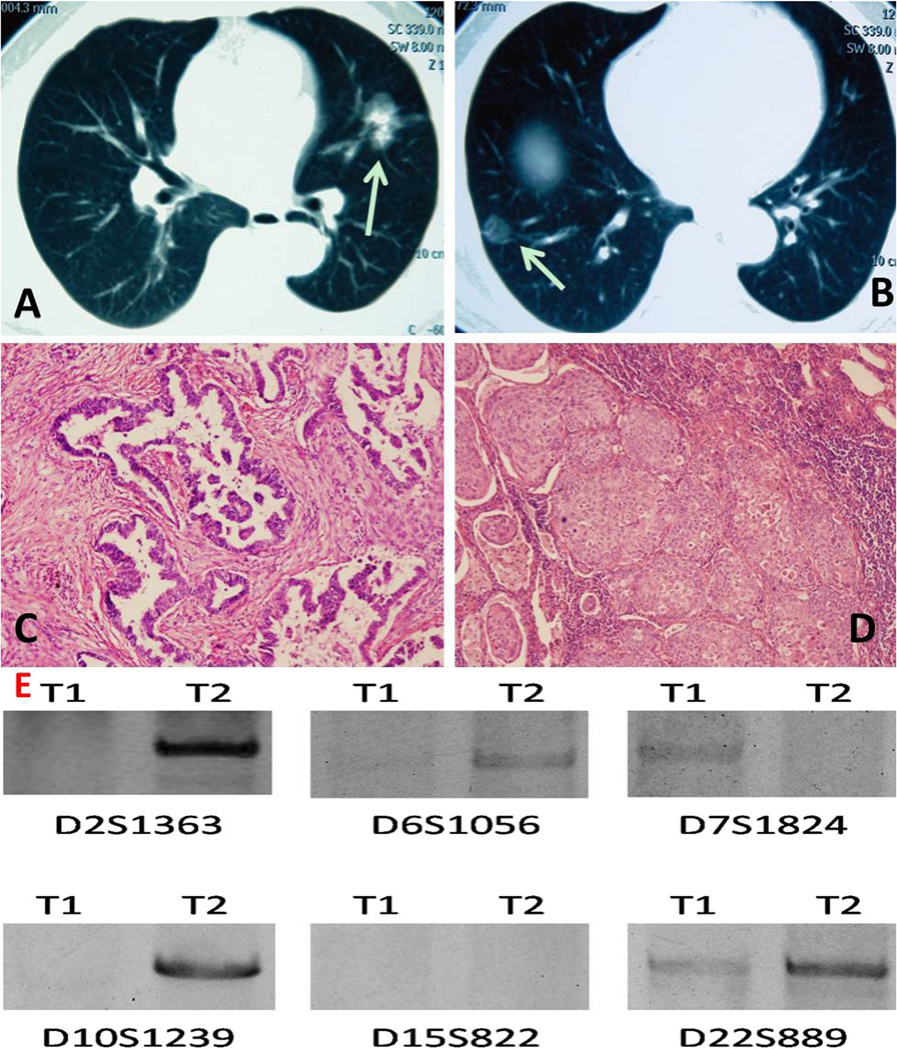
**CT features, histological features, and molecular analysis of patient 5 in metachronous lung tumor group.** CT features, histological features, and molecular analysis of patient 5 in the metachronous lung tumor group of different histological types. **A** and **B**: the first tumor and the second tumor of patient 3 in the metachronous lung tumor group of different histological types. **C** and **D**: the first tumor and the second tumor were diagnosed respectively as squamous carcinoma and adenocarcinoma (H & E staining X100). **E**: T1 refers to the first tumor in the left upper lobe, and T2 refers to the second tumor in the right lower lobe.

All the patients had undergone a surgical resection between April 2003 and December 2013 in the Department of Thoracic Surgery at West China Hospital, Sichuan, China. Five patients were diagnosed with multiple primary lung cancers according to criteria proposed by Martini and Melamed [17], and seven patients were diagnosed as intrapulmonary metastasis. The five patients were classified as metachronous tumors with or without the same histological type. Among them, two patients were diagnosed as having multiple primary lung cancers with the same histological types, and three patients were diagnosed as having different histological types. All patients were followed up and their status recorded as alive or dead. The first tumor was designated as tumor 1 (T1), while subsequent tumors were designated as tumor 2 (T2). The age, site of tumor, histology, sex, and the status of following up for each patient are given in Table 1 and Figures 1 and 2. The experiments were approved by the West China Hospital Ethics Committee (No.2013 (33)), and all patients agreed to participate in the study and signed an informed consent form.

**Table 1:** **Clinical, pathologic data and microsatellite marker of all patients**

**Patients**			**Age**	**Sex**	**Site of tumor**	**Histology**	**Interval (months)**	**Follow-up**	**D2S1363**	**D6S1056**	**D7S1824**	**D10S1239**	**D15S822**	**D22S689**
*G1*	*P1*	T1	41	F	RLL	AC	29	Alive	+++	++	+	+	-	-
		T2			RUL	AC			-	++	+	+	-	-
	*P2*	T1	71	M	RUL	AC	42	Dead	+	-	+	-	-	-
		T2			RML	AC			-	-	-	+	-	-
*G2*	*P3*	T1	72	M	RLL	SCC	40	Dead	++	-	+	+	-	-
		T2			LUL	AC			-	+	+	-	-	-
	*P4*	T1	63	M	RML	AC	12	Alive	-	+	+	++	+	-
		T2			RUL	SCC			+	+	+	-	-	-
	*P5*	T1	60	M	LUL	SCC	17	Alive	-	-	+	-	-	+
		T2			RLL	AC			+	++	-	+	-	+
	*P6*	T1	62	M	LLL	SCC	None	Alive	+	-	+	+	++	+
*G3*		T2			LUL	SCC			+	-	+	+	-	-
	*P7*	T1	65	M	LUL	AC	None	Alive	-	-	+	++	-	+
		T2			LLL	AC			-	-	-	++	-	-
	*P8*	T1	55	F	RUL	AC	None	Alive	+	+	+	-	-	+
		T2			RML	AC			-	-	+	-	-	-
	*P9*	T1	66	M	RUL	AC	None	Dead	-	-	+	+	-	-
		T2			RLL	AC			**+**	++	+	+	+	-
	*P10*	T1	51	F	RUL	AC	None	Alive	++	++	+	+	+	+
		T2			RLL	AC			++	+	+	-	+	+
	*P11*	T1	40	F	RUL	AC	None	Alive	+	-	+	+++	-	-
		T2			LLL	AC			+	+	+	++	++	+
	*P12*	T1	56	M	LUL	SCC	None	Alive	+	+	+	+	-	-
		T2			LLL	SCC			+	-	+	-	-	-

T1 refers to the first neoplasm; T2 refers to the second neoplasm.

G1, metachronous lung tumor with same histological types; G2, metachronous lung tumor with different histological types; G3, intrapulmonary metastasis lung tumors; AC, adenocarcinoma; SCC, squamous carcinoma; RUL, right upper lobe; RML, right middle lobe; RLL, right lower lobe; LUL, left upper lobe; LLL, left lower lobe.

### Preparation of genomic DNA

Sections stained with hematoxylin and eosin (H & E) were used to identify regions of well-preserved tumor tissue containing ≥90% tumor. Tumor tissues from three to five unstained 6-μm sections were dissected free of tumor stroma using a sterile scalpel for each case and collected in 600 μl of xylene. Deparaffinization was achieved by incubating for 5 min in xylene, followed by centrifugation at 13,000× g for 4 min to collect deparaffinized tissues. The tissue pellet was resuspended in 600 μl of 95% ethanol, washed for 6 min, and centrifuged at 13,000× g for 5 min. The resulting tissue pellet was dried, and DNA was isolated using a paraffin-embedded tissue genomic DNA extraction kit (Bioteke Corporation, Beijing, China) according to the manufacturer’s protocol.

### Microsatellite PCR analysis

Genomic DNA collected from tumor samples were examined for six polymorphic microsatellite markers including: D10S1239, D7S1824, D2S1363, D6S1056, D15S822, and D22S689. The oligonucleotide primers corresponding to each microsatellite marker were purchased from Sangon Biotech (Shanghai, China) Co., Ltd. For PCR, the reaction samples were each prepared to a total volume of 25 μL as follows: 1 μL each primer, 1 μL template DNA, 9.5 μL nuclease-free water, and 12.5 μL PCR Mix (2X). Amplifications were carried out in a Perkin Elmer Thermocycler (Perkin Elmer, Waltham, USA) using a step-cycle program consisting of 39 cycles of 95°C for denaturing (30 s), 55°C for annealing (30 s), and 72°C for extension (1 min). PCR products were fractionated on 6% polyacrylamide gels containing Tris-borate/EDTA (pH 8.0) and visualized by ethidium bromide staining.

## Results

### Analysis of metachronous lung tumor with same histological types

Two cases presented with metachronous multiple tumors of the lung were studied for molecular analysis. The microsatellite PCR results for each of these patients are given in Table 1.

Patient 1 was diagnosed with adenocarcinoma of the right lower lobe at the first time. Twenty-nine months later, a newly node was found in the right upper lobe. In this case, the molecular evidence was based upon disconcordant results with D2S1363. Allele corresponding to microsatellite marker D2S1363 was observed in T1 but was not detected in T2.

Molecular analysis of patient 2 provided a clear example of a patient as having metachronous lung tumor. Alleles for D2S1363 and D7S1824 were detected in the T1 that were not observed in the subsequent lung tumor (T2). In contrast, allele corresponding to D10S1239 was detected in the lung cancer (T2) that was not observed in the T1.

### Analysis of metachronous lung tumor with different histological types

Three cases of metachronous tumors with different histological types (Table 1) of the lung were also studied for molecular analysis. In the typical case of patient 5, the first neoplasm was found in the left upper lobe, which was pathologically diagnosed as squamous cell carcinoma after a successful surgical lobectomy. Seventeen months later, newly ground-glass opacity was observed in the right lower lobe by CT (Figure 2). Then, the patient underwent a pulmonary wedge resection and the pathology was adenocarcinoma (Figure 2). In our study, the observed allelic variation of patient 5 at D2S1363, D6S1056, and D10S1239 suggests that T1 could be derived from T2, consistent with dual primary tumors (Figure 2 and Table 1). However, the allelic variation noted at D7S1824 contradicts this possible lineage relationship.

In the other two patients, alleles corresponding to microsatellite markers D2S1363 and D10S1239 were amplified in DNA from T1 of patient 3 but were not observed in DNA from T2. However, in the same patient, D6S1056 was detected in DNA from T2 but was not found in DNA from T1. Similar results were obtained for patient 4.

### Analysis of intrapulmonary metastasis lung tumor

Seven pairs of tumors were chosen, each consisting of a “primary lung tumor” and a “metastatic tumor” (Table 1). Lineage relationships for each of these tumor pairs were determined by molecular analysis.

For example, alleles corresponding to microsatellite markers D2S1363, D6S10556, and D22S689 were detected in DNA from T1 of patient 8 whose provided a clear example of a patient with clonally related neoplasms but were not observed in DNA from T2 (Figure 1 and Table 1). Likewise, alleles corresponding to microsatellite markers D7S1824 and D22S689 were observed in T1 of patient 7 but were not detected in T2. Similar results were obtained for patients 6, 10, and 12. In the case of patient 9, which the tumor designations were assigned arbitrarily when multiple tumors were surgically removed from a given patient at the same time, represented inverted consequence in the Table 1. Alleles for D2S1363, D6S1056, and D15S822 were observed in T2 but were not detected in T1.

**Figure 3:**
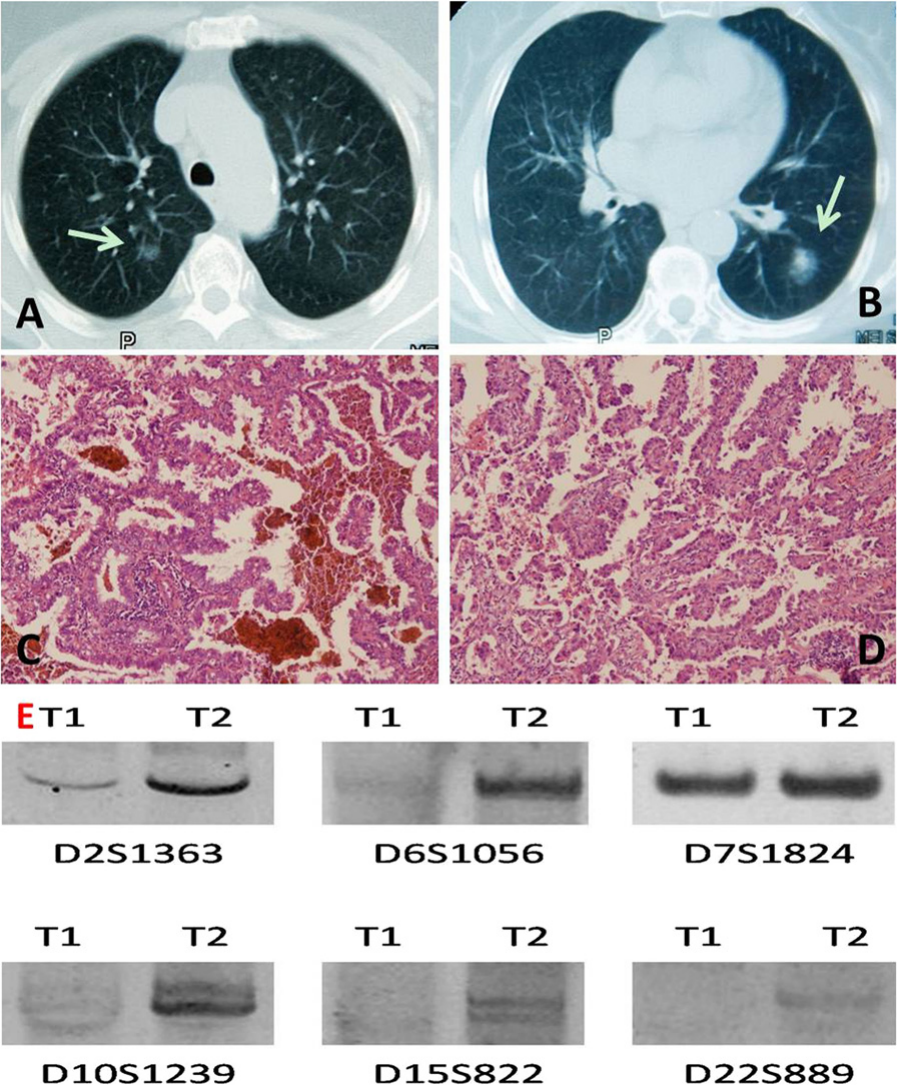
**CT features, histological features, and molecular analysis of patient 11 in the intrapulmonary metastasis group. A** and **B**: the first tumor and the second tumor of patient 11. **C** and **D**: two neoplasms in different chest of patient 11 in the intrapulmonary metastasis group were diagnosed pathologically as adenocarcinoma (H & E staining X100). **E**: T1 refers to the tumor in the right upper lobe, and T2 refers to the tumor in the left lower lobe.

In the case of patient 11 (Figure 3 and Table 1), the two neoplasms were found in the left lower lobe and right upper lobe at the same time by CT. She was a non-smoker and had no exposure to any environmental fumes or dust. Physical examination revealed normal breath sounds in both of the lung fields. Laboratory findings were within normal limits. Her pulmonary function tests and cardiovascular examination revealed normal performance. According to the fundamental state of all the examinations, the patient underwent a successful left lower lobectomy and right upper pulmonary wedge resection at the same time. The pathology of two tumors was adenocarcinoma. The patient was followed up without evidence of recurrence to date. In our study, the two tumors share common allelic patterns for two microsatellite marker at D2S1363 and D7S1824 but show differing allelic patterns for four microsatellite markers at D6S1056, D10S1239, D15S822, and D22S689 (Figure 3 and Table 1). Alleles corresponding to microsatellite markers D10S1239 were observed in T1 but were not detected in T2. At the same time, microsatellite markers D6S1056, D15S822, and D22S689 were observed in T2 but were not detected in T1.

## Discussion

It may be easy to diagnose multicentric primary lung cancers in multiple lung lesions when their histological types are different. However, if they show the same histological type, it is often difficult to discriminate multiple primary lung cancers from intrapulmonary metastasis.

The differential diagnosis of intrapulmonary metastatic lung cancer (via bloodstream dissemination) verse primary lung cancer is also perplexing in clinical setting; however, tumors with consistent histology and N2 and N3 lymph nodes or multiple-organ metastasis are often diagnosed as intrapulmonary metastasis [4].

On the basis of the analyses of the overall survival among pathologically staged cases, nodules within the same lobe as the primary lesion are categorized by the International Union Against Cancer (UICC) as T4 and those located in the different lobe (whether it is ipsilateral or contralateral side) as M1. The International Association for the Study of Lung Cancer (IASLC) lung cancer-staging project recommended changes in the T classification to subclassify additional nodules in the same lobe as T3, nodules in the ipsilateral different lobe as T4, and those in a contralateral lobe as M1 [18]. Their different biologic behaviors may, thus, be responsible for prognostic differences. Because some of the patients with intrapulmonary metastasis show poor prognosis, it is necessary to discriminate intrapulmonary metastasis from multiple primary lung cancers by exploring new practical techniques and markers.

The pathogenic mechanism of multiple primary lung cancer is still unclear, but several authors have reported the feasibility of clonal analyses between tumors to discriminate multiple primary lung cancers from intrapulmonary metastasis. A multiple-gene analysis to identify the clonality in a combination of multiple-gene mutations, such as a p53 gene mutation, K-ras mutation, and/or loss of heterozygosity, has been reported [19-24]. Mitsudomi analyzed the p53 gene mutation in 16 patients with multiple primary lung cancers [25]. Among those patients, seven were not informative because of the absence of the p53 mutation in both tumors. Among nine patients who had at least one p53 mutation in their pair of tumors, six were suggested to be of different clonal origin and were diagnosed with multiple primary lung cancers. Matsuzoe reported that 7 of 20 patients who were clinically diagnosed to have intrapulmonary metastasis showed different p53 gene mutations between the two lesions, thus indicating these lesions to be multiple primary lung cancers [26]. These reports indicate that the somatic mutations of the p53 gene may be a suitable biological factor to identify multiple primary lung cancers. Although there is a positive expression of the p53 due to a prolonged half-life time induced by a somatic mutation of the p53 gene, the concordance rate between p53 gene mutations and immunopositivity in NSCLC is reported to be 60% to 70% [27,28]. According to Ono’s study, the sum value of the differences in the expression ratio of four proteins (p53, p16, p27, and c-erbB2) was evaluated in immunohistochemically stained specimens among multiple primary lung cancers and intrapulmonary metastasis [29]. Yoshimoto reported a case, diagnosed as double primaries, that is distinguished by EGFR gene mutation analysis [30].

Mercer et al. [15] found that detections of microsatellite alterations and deletion sites in tumor cell DNA could be used as diagnostic and prognostic markers for multiple cancers. In our previous study [16], the results demonstrated that molecular analysis of allelic variations at polymorphic microsatellite markers can be used to determine lineage relationships between multiple tumors, facilitating the discrimination of second primary cancer versus metastatic disease. With polymorphic microsatellite markers, the “unique trend” that represents metastasis cancers and the “contradictory trend” that represents primary multiple tumors are useful in the diagnosis between tumors found at the same time in the pulmonary even diagnosed with the histopathological evaluation.

In the first group, as the consequences showed of two patients in our study, alleles in patient 2 corresponding to microsatellite markers D2S1363 and D7S1824 were in the DNA from the first tumor but were reduced or not observed in the DNA from the second. In contrast, allele corresponding to D10S1239 was detected in the lung cancer (T2) that was not observed in the T1. Molecular analysis of these tumors identified discordant allelic variations involving three different microsatellite markers, arguing that these cancers arose independently. These mutually exclusive allelic losses strongly suggest that both of the neoplasms are not related. Thus, the lung cancer in this patient represents a second primary carcinoma rather than a solitary metastatic lesion derived from the other one. The characteristic of this “contradictory trend” is representative as our study before. However, in the case of patient 1, the two tumors share common allelic patterns for five microsatellite markers but show differing allelic patterns for only one microsatellite marker at D2S1363. The allele corresponding to the microsatellite marker D2S1363 was observed in T1 but was not detected in T2. The results strongly suggest that the T1 has given rise to T2 and suggest that the T1 metastasized to the T2. Given the histopathology for these neoplasms, a conclusion that a metastatic lesion derived from the other one was the same as ours. The “unique trend” is made with less confidence when the numbers of discordant changes are this few. Given that there is a concern about the effects of intra-tumor heterogeneity on this type of analysis, increased numbers of microsatellite markers (and observations of allelic variation) might be required to draw clear conclusions.

The next groups of tumors we studied were metachronous tumors with different histological types of the lung, and the result showed a “contradictory trend”. The paired tumors in case 3 appeared to be typical of the three patients. The observed allelic variation at D7S1824 suggests that T2 could be derived from T1, consistent with metastatic disease. However, the allelic variations of D2S1363, D6S1056, and D10S1239 were noted in T2 but not observed in T1; which means that T1 could be also derived from T2, so that the result contradicts the possible lineage relationship of metastatic disease.

Among the third group including seven intrapulmonary metastasis lung tumors diagnosed by pathology, the results of cases 6, 7, 8, 9, 10, and 12 present typical “unique trend” and it suggests that all the patients represent the real intrapulmonary metastasis lung tumors. However, the controversial consequences happened in patient 11. As the result showed before, patient 6 provided a clear example of a patient with two unrelated tumors, where the consequence suggests a “contradictory trend”. In this case, microsatellite markers D6S1056, D15S822, and D22S689 were observed in T2 but were not detected in T1. At the same time, alleles corresponding to microsatellite marker D10S1239 was observed in T1 but was not detected in T2.

## Conclusion

It is important to discriminate intrapulmonary metastasis from multiple primary lung cancers. However, in some cases, such discrimination can be difficult. Although new methods have recently been applied to this differentiation, they are not practical at present. The results of this study suggest that not only immunohistochemical analyses of differential protein expression profiles of multiple genes can be used but also molecular analysis of allelic variations at polymorphic microsatellite markers to distinguish multiple primary lung cancers from intrapulmonary metastasis. The “unique trend” that represents metastasis cancers and the “contradictory trend” that represents primary multiple tumors are useful in the diagnosis between tumors even diagnosed with the histopathological evaluation.
